# Effect of *Melissa officinalis* L. Essential Oil Nanoemulsions on Structure and Properties of Carboxymethyl Chitosan/Locust Bean Gum Composite Films

**DOI:** 10.3390/membranes12060568

**Published:** 2022-05-30

**Authors:** Huijie Yu, Chi Zhang, Yao Xie, Jun Mei, Jing Xie

**Affiliations:** 1 College of Food Science & Technology, Shanghai Ocean University, Shanghai 201306, China; m210300835@st.shou.edu.cn (H.Y.); m210300872@st.shou.edu.cn (C.Z.); m210311052@st.shou.edu.cn (Y.X.); 2 Shanghai Professional Technology Service Platform on Cold Chain Equipment Performance and Energy Saving Evaluation, Shanghai Ocean University, Shanghai 201306, China; 3 National Experimental Teaching Demonstration Center for Food Science and Engineering, Shanghai Ocean University, Shanghai 201306, China; 4 Shanghai Engineering Research Center of Aquatic Product Processing & Preservation, Shanghai Ocean University, Shanghai 201306, China

**Keywords:** essential oil, active film, nanoemulsions, antibacterial activity

## Abstract

This study aimed to develop active films based on carboxymethyl chitosan (CMCS)/locust bean gum (LBG) films containing *Melissa officinalis* L. essential oil (MOEO) nanoemulsions. The results showed that the active films incorporated with MOEO nanoemulsion resulted in an increase in the elongation of break, water resistance and improved the film hydrophilicity. Elongation of break increased from 18.49% to 27.97% with the addition of 4% MOEO nanoemulsion. Water resistance was decreased from 56.32% to 25.43%, and water contact angle was increased from 75.13 to 83.86 with the addition of 4% MOEO nanoemulsion. However, the water vapor barrier properties and tensile strength decreased with the addition of MOEO nanoemulsions. The scanning electron microscopic images and Fourier transform infrared spectroscopy results showed that the MOEO was very compatible with the film materials and dispersed evenly in the films. At the same time, the addition of MOEO nanoemulsion significantly enhanced antioxidant and antibacterial activities of C/L-MOEO films. The antioxidant and antimicrobial activities of C/L-MOEO films were increased from 7.16% to 33.81% and 3.52% to 54.50%, respectively. In general, C/L-MOEO film has great application prospects.

## 1. Introduction

The accumulation of polymeric and petroleum-based packaging materials causes serious environmental pollution and poses a threat to human health [1]. In addition, the question of food spoilage caused by microorganisms cannot be resolved. The development of bio-based antibacterial active packaging highlights a new trend in the food industry [2]. The bio-based antibacterial active packaging is prepared by incorporating antibacterial substances such as natural bacteriostats or chemical preservatives into bio-based matrix. The addition of antibacterial substances can maintain the quality of food, prolong shelf-life, and inhibit deterioration of the film, simultaneously [3]. For example, high amylose starch (HAS)-konjac glucomannan (KGM) mixed with cinnamaldehyde, a new bio-based antibacterial active packaging, improved the hygroscopicity and perishability of film and endowed it with the antibacterial ability of HAS-KGM film, simultaneously [3].

Locust bean gum (LBG), a vegetable gum extracted from the seeds of the carob tree, has the potential to be a futuristic biopolymer because of its versatile applications in water purification, additives (E410), gelling, excipients for pharmaceuticals, stabilizers, emulsifying agents and food packaging [4,5]. The LBG–chitosan (CS) based film has been used in food packaging due to its excellent formability [6]. CS is a natural linear polycationic polysaccharide that can be developed for potentially functional film applications due to its antimicrobial, biodegradable and exceptional film-forming characteristics [7]. Despite these beneficial features, the practical applications of CS are largely hindered due to its poor mechanical properties and poor solubility in water due to the presence of a hydrophobic acetyl group [8]. Whereas CS has poor solubility in water, carboxymethyl chitosan (CMCS) has good water solubility and retains the advantages of CS [9], making it a promising candidate to replace CS. Therefore, CMCS and LBG have potential as film matrixes with broad applications in the packaging industry.

*Melissa officinalis* L. is a perennial herbaceous plant belonging to the Lamiaceae family [10]. The leaves of this plant contain monoterpenes with anti-apoptotic, antioxidant and anti-amyloidogenic effects and are usually applied in the form of powdered herbal substances, herbal teas, or essential oil [11]. The main ingredients in *Melissa officinalis* L. essential oil (MOEO) are phenolic components and terpenes with antioxidant and antibacterial activity, making it one of the most attractive essential oils. The addition of MOEO into bio-based film provides a new idea for the application of MOEO. However, MOEO is water insoluble and highly volatile, and should be prepared as an essential oil emulsion and then dispersed into film-forming solutions to increase the retention of the essential oil. One of the important impacts on the properties of active films is the droplet size of the essential oil emulsion, which affects the stability of oil-in-water emulsions. High-intensity ultrasonic homogenization can reduce the droplet size of coarse emulsions by homogenizing the diaphasic systems [12].

The object of this research was to prepare C/L based active films incorporated with MOEO nanoemulsions. The effects of MOEO nanoemulsions on the microstructure, mechanical properties, hydrophobicity, water vapor barrier (WVP), transmittance, oxygen transmission rate (OTR), and antioxidant and antibacterial properties of C/L-MOEO films were investigated.

## 2. Materials and Methods

### 2.1. Materials

CMCS was supplied by Shanghai Macklin Biochemical Co., LTD (Shanghai, China), with degree of deacetylation >90%, isoelectric point: 3–4. LBG (molecular weight: about 300,000 Da) and Tween-80 were purchased from Aladdin Biochemical Technology Co., LTD (Shanghai, China). All other chemical agents were of analytical grade and all solutions were formulated with deionized water.

### 2.2. Fabrication of MOEO Emulsions

MOEO was extracted using a Clevenger type apparatus. The leaves were washed to remove the dirt and were subjected to hydro-distillation for 6 h. The essential oil was then collected and stored in a dark glass vial at 4 °C. The preparation of MOEO nanoemulsions referenced the method from Ghosh et al. [13]. Tween-80 was used as an emulsifier to stabilize the W/O emulsion. MOEO and Tween-80 (10% MOEO, *v*/*v*) were added to the deionized water, and the mixed coarse emulsion was treated with an ultrasonic homogenizer at 20 kHz, 750 W (Xiecheng Ultrasonic Equipment Co., Ltd., Jining, China) for 15 min in pulsed mode (30 s ON and 30 s OFF) with a 13 mm diameter sonicator probe to obtain the nanoemulsion [14].

### 2.3. Preparation of Active Films

**Figure 1:**
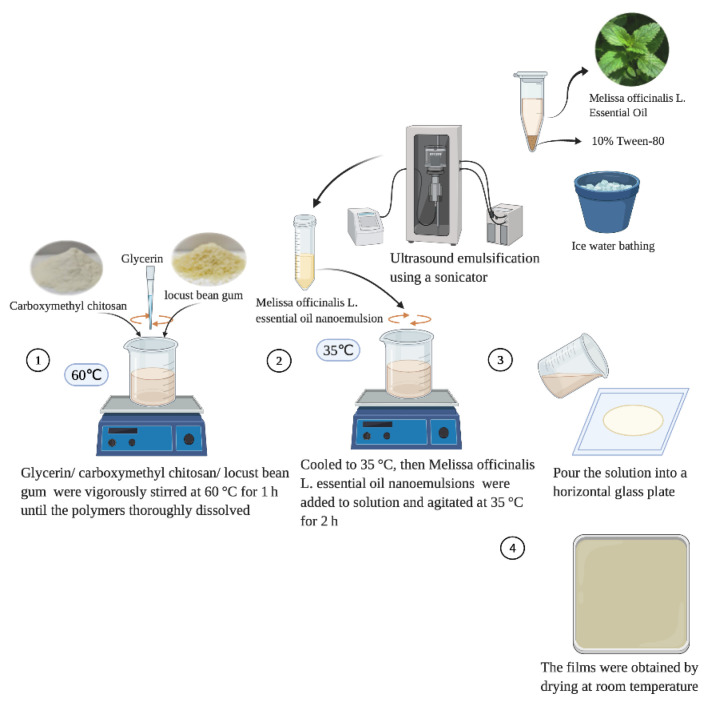
The preparation of carboxymethyl chitosan/locust bean gum/*Melissa officinalis* L. essential oil active film.

The preparation of C/L active film referenced the method from Keawpeng et al. [15]. As shown in Figure 1, 1 g of CMCS and 1 g of LBG were incorporated in 200 mL deionized water with 0.4 g of glycerol (20% gly/CMCS/LBG, *w*/*w*) added as a plasticizer and vigorously stirred at 60 °C for 1 h until the polymers thoroughly dissolved. When the mixture emulsion was cooled to 35 °C, MOEO nanoemulsions were incorporated, and the final concentrations of MOEO were 1, 2 and 4 μg/mL, respectively. The final mixture emulsion was agitated at 35 °C for 2 h. At the end, air bubbles were removed by vacuum. A 200 mL film solution was poured into a horizontal glass plate (25 cm × 25 cm) and dried (22 °C, 50% relative humidity). The dried films were stripped from the glass plates and conditioned at 25 °C and 50% R. H. for at least 72 h. The films were named as C/L, C/L-1%M, C/L-2%M and C/L-4%M, respectively, according to the concentration of MOEO.

### 2.4. Characterization of Emulsion

#### 2.4.1. Dynamic Light Scattering (DLS)

The droplet size of the emulsions was determined with a laser diffraction particle size analyzer (Beckman Coulter, Inc., Brea, CA, USA). The Brownian motion of nanoscale droplets resulted in a scattering light intensity change hat be detected by DLS technique [16].

#### 2.4.2. Transmission Electron Microscopy (TEM)

The diluted MOEO nanoemulsions were deposited over a copper grid with a carbon film, and then dried. Emulsion droplet morphology was observed by TEM (model JEOL JSM-100 CX, Shimadzu Co., Tokyo, Japan) [16].

### 2.5. Physical and Mechanical Properties of Active Films

#### 2.5.1. Thickness

Twelve points were randomly measured on the films by a helical micrometer (Mitutoyo, Shanghai, China) with an accuracy of 0.001 mm, and the average was treated as the film thickness [17].

#### 2.5.2. Moisture Content and Water Solubility

The moisture content of the films was determined by the gravimetric method after being dried at 105 °C for 24 h [17].

The water solubility of the films was measured by the method of Mei et al. [18] with some modifications. A square section of film (20 × 20 mm) was weighed to obtain the initial dry weight (M_1_). The films were dipped in 100 mL of deionized water at room temperature for 30 min. Undissolved films were taken out and dried in an oven at 105 °C until constant weight, which was recorded as M_2_. Water solubility was calculated with the following Equation (1).

(1) $$\mathrm{Water}\;\mathrm{solubility}=\frac{M_{1}-M_{2}}{M_{1}}\times 100\%$$

#### 2.5.3. Mechanical Properties

The mechanical properties of the films were measured by the method from Wang et al. [19]. A high-precision double-blade cutter was used to cut films into rectangular strips (2 × 15 cm). The tensile strength (TS) and elongation at break (EAB) of the films were determined with a TA-XT2i texture analyzer (Stable Micro Systems, Surrey, UK). The initial grip separation was 50 mm, and the probe speed was 1 mm/s.

#### 2.5.4. Water Vapor Permeability (WVP)

The WVP of active films was estimated with a WVP tester (Labstone, W-B-31E, China). Circular films were used to seal test dish cups injected with identical amounts of deionized water and tested in a desiccator (38 °C and 10% R.H.) [15].

#### 2.5.5. Oxygen Barrier Properties

The oxygen transmission rate (OTR) of active films was estimated with a gas permeability tester (Labthink, G2/132, China) [15]. The films were placed between the top and bottom cups in a test chamber containing oxygen. The tests were run at 23 °C.

### 2.6. Optical Properties

#### 2.6.1. Color

The color of films was estimated with a Chroma meter (Konica Minolta, CR-400, Tokyo, Japan) to measure L*, a*, b*. ΔE and chroma were calculated with the following Equations (2) and (3), respectively [19]. The value was the average of three random points on each film.

(2) $$\Delta E=\sqrt{\Delta L^{*}{}^{2}+\Delta a^{*}{}^{2}+\Delta b^{*}{}^{2}}$$

(3) $$C=\sqrt{a^{*}{}^{2}+b^{*}{}^{2}}$$

#### 2.6.2. Opacity

The light barrier properties of the films were investigated with a UV-visible spectrophotometer (HITACHI, U-3900, Japan) using the method of Agarwal et al. [20]. Opacity was determined by measuring each film's transmittance spectra at 600 nm with air as a blank.

### 2.7. Contact Angle Measurements

The contact angles of the active films were determined with a contact angle instrument (SDC-100, SINDIN, China) [21]. The sample films were cut into strips of 50 mm × 10 mm. A 3 μL amount of deionized water was dropped onto the films through an automatically controlled microinjector. The obtained value was the average of measurements at three random points on each film.

### 2.8. Scanning Electron Microscopy (SEM)

The films were fractured in liquid nitrogen. A gold layer was sputter-coated on films. The surface and cross profile micrographs of the active films were obtained in a Hitachi S-5000 N scanning electron microscope at 5.00 kV operating voltage [22].

### 2.9. Fourier Transform Infrared Spectroscopy (FTIR)

FTIR spectra of the active films were measured with an ATR/FT-IR spectrometer (Thermo Fisher, Nicolet iS5, USA) within 650–4000 cm^−1^ at a resolution of 4 cm^−1^ and 64 scans [22].

### 2.10. Characterization of Antioxidant and Antibacterial Properties

#### 2.10.1. DPPH Radical Scavenging Assay

Antioxidant activities of the active films were assessed by DPPH radical scavenging rate test [22]. DPPH was dissolved in 4 mg/mL methanol, and 250 mg of the films were incorporated into the 10 mL methanolic solution of DPPH and incubated for 30 min at 30 °C. The absorbance was measured at 517 nm. The control was a methanolic solution of DPPH without film samples.

#### 2.10.2. Antibacterial Properties

The antibacterial properties of films against *Shewanella putrefaciens* were investigated by the method of plate counting [23].

### 2.11. Statistical Analysis

All measurements were performed in triplicate. Data were expressed as the average ±SD. All statistical analyses were performed using Prism 8.

## 3. Results

### 3.1. Characterization of MOEO Nanoemulsion

**Figure 2:**
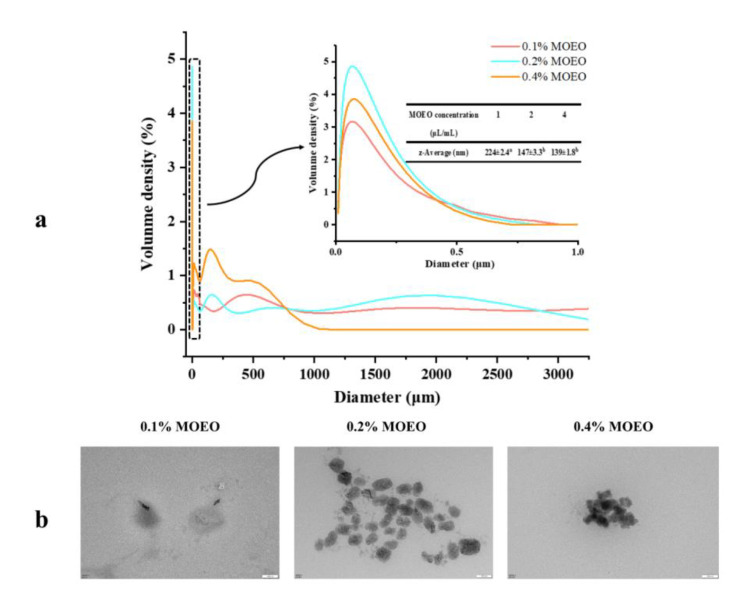
(**a**) Droplet size distribution and (**b**) transmission electron microscopy of *Melissa officinalis* L. essential oil nanoemulsions.

The mechanical, physical and optical properties of the films were affected by the droplet size of the nanoemulsion [7]. The droplet size distribution was tested by DLS (Figure 2a). The average droplet sizes for C/L-1%M, C/L-2%M and C/L-4%M were 224 nm, 147 nm and 139 nm, respectively. The stability of the emulsions was attributed to the ultrasonic homogenization, because the cavitation bubbles in the liquid were caused by ultrasound waves [24]. Then, the energy of breaking oil droplets was obtained from the collapsed bubbles on the surface of essential oil droplets [24]. The nano-droplets were stabilized by the surfactant molecules (Tween 80 in the present research), and the steric hindrance reduced the degree of collision between droplets [25].

Figure 2b shows the TEM images of the emulsions. The average sizes of the droplets were approximately 200 nm, 100 nm and 80 nm for C/L-1%M, C/L-2%M and C/L-4%M, respectively, which were smaller than the results obtained with DLS. DLS measured the hydrodynamic radius of a nanodrop surrounded by solvent molecules, resulting in a relatively larger size [15].

### 3.2. Physical Properties of Films

#### 3.2.1. The Thickness of Films

Compared to C/L film, the incorporation of MOEO nanoemulsion caused a considerable increase in thickness (Table 1). Some researchers also reported that the incorporation of plasticizers and lipid materials could increase the thickness of films. Emulsification with an appropriate surfactant/oil ratio and application of sufficient acoustic energy increased the stability of the film-forming solution when drying; thus, the loss of MOEO decreased, and the thickness of films increased [26]. At the same time, micro-droplets formed from the hydrophobic MOEO during homogenization of the film-forming solution also increased the thickness of film [27]. Moreover, the increasing concentration of MOEO nanoemulsion increased the total solid content, resulting in the increasing thickness of film.

#### 3.2.2. Mechanical Properties

TS is the maximum force per unit area that the film withstood before being fractured, while EAB is the measured ratio of the extension of the active film to the initial length before being fractured. The presence of MOEO nanoemulsion within the film matrix reduced TS and increased EAB (Table 1). The decrease in TS primarily due to the intra- and inter-molecular interactions was reduced by the penetration of MOEO into the biopolymer network. The presence of MOEO nanoemulsion replaced the strong intermolecular polymeric interactions with weak interactions of the polymer–oil matrix in the film. This phenomenon was related to the heterogeneous coalescence and dispersion of MOEO in the matrix of C/L caused by large size droplets. The increase in EAB indicated that the active films were more stretchable and flexible due to the incorporation of MOEO nanoemulsion with a strong plasticizing effect that caused the polymer chains to move and promote the flexibility of films [28]. The increase in EAB was positively related to the concentration of the MOEO nanoemulsion. This discovery was consistent with Otoniet al. [29], who reported that the decrease in droplet size could improve the plasticizing effect, causing films have a higher EAB. In addition, the thicker films required more strength to be broken and had higher elongation [30]. The films with higher thickness were observed to be more resistant to fracture because the denser matrix of polymer was strengthened by high-level molecular interactions.

#### 3.2.3. WVP and Water Contact Angle

The WVP of the active films is important in food packaging because the shelf life can be affected by the transfer of water vapor into the packaged food [31]. The WVP of active films was 7.47 × 10^−12^ g cm/cm^2^ s Pa in C/L and 8.67 × 10^−12^ g cm/cm^2^ s Pa in C/L-4%M (Table 1). The increased WVP was mainly because the MOEO emulsion generated micropores and cavities in the films' structure, resulting in the diffusion of water vapor molecules [27]. The above results could also be observed in the SEM images. Bonilla et al. [32] also showed that the cohesion was reduced because of the discontinuity of the polymer network due to MOEO nanoemulsion. Similar trends were found in CMCS–pullulan active films containing galangal essential oil and in xanthan gum/locust bean gum films containing tea polyphenols [33].

The water contact angle evaluated the wettability on the surface of active films, and the results are shown in Table 1. All films had a water contact angle of less than 90°, indicating that they were hydrophilic. The addition of MOEO nanoemulsion expressively enhanced the water contact angle of the films, but they were still hydrophilic. The increase in water contact angle of the C/L films was mainly due to the hydrophobicity of MOEO. Wu et al. [34] added oregano essential oil to cellulose nanofibril films, increasing the water contact angle of the film from 55.5 to 73.7 owning to the hydrophobic groups in the essential oil. In addition, Chen et al. [35] decreased the hydrophilicity of films by adding cinnamaldehyde to starch/polyvinyl alcohol films.

#### 3.2.4. Moisture Content and Solubility

The C/L had the highest moisture content (19.59%, Table 1), and the incorporation of MOEO nanoemulsion caused the moisture content of the active films to decrease (Table 1). The MOEO nanoemulsion had hydrophobic dispersity and could increase the hydrophobicity of films. Similar results were observed by Peng et al. [36] and Ojagh et al. [37], who added hydrophobic compounds to chitosan active films. However, there were no significant differences between the films and those with MOEO emulsions (*p* > 0.05). In addition, when MOEO dried at 105 °C, it evaporated with water vapor and the water content might have been slightly overestimated. The water resistance or insolubility for active films is also important, particularly in humid environments [4].

Lower water solubility of films materials can extend the shelf life of food by maintaining the water resistance of films [38]. Film water solubility was negatively correlated with the concentration of MOEO nanoemulsions (*p* < 0.05), which might be due to the decrease in hydrophilicity of the films after the hydrophobic MOEO emulsion was incorporated. Some studies also showed that the addition of hydrophobic materials to films reduced their water solubility [39]. Moreover, the interaction of hydroxyl groups with water molecules was reduced because the addition of essential oil reduced the solubility of the films [40].

#### 3.2.5. Oxygen Barrier Properties

Oxygen is an essential environmental factor that can lead to the spoilage of food during storage. The oxygen barrier capacity increased significantly with the addition of MOEO nanoemulsions (Table 1). This might be because of the emulsification effect of ultrasonic treatment, which improved the homogeneity of MOEO in the films and avoided phase separation within the system. In addition, the compatibility of the film matrix with MOEO nanoemulsions caused a dense structure, making it more difficult for non-polar oxygen molecules to cross the film. Furthermore, the antioxidant activity of MOEO nanoemulsions provided sufficient free radical scavenging activity and positively influenced the oxygen barrier properties of the films [41]. However, the oxygen barrier capacity of the films was reduced because the micropores increased, which was caused by the increased volatilization of MOEO.

### 3.3. Color Properties

The color of active films affects the appearance and consumer acceptance of packaged products [42]. In Table 2, the concentration of MOEO nanoemulsion did not show a significant effect on greenness (a*). The yellowish color was an intrinsic property of CMCS [43]. The yellow color of MOEO emulsion also led to a significant increase in the yellowness (b*) of the active films. The lightness (L*) decreased with the addition of MOEO nanoemulsion probably because the phenolic components in MOEO caused refraction of light and scatter, resulting in darker films [44]. Increased concentrations of MOEO nanoemulsion resulted in increases in lightness (L*) and total chromatic films. Similar results were reported by Kadam et al. [45] and Souza et al. [46], who found that chitosan films with added polyphenolic extracts of Nigella sativa seedcake and extract of mango leaf increased lightness (L*), and total chromatic films were all increased. The results indicated that the addition of MOEO nanoemulsions could make the films yellow and darker, and the color of active films was the same as the coloring components in the MOEO.

The opacity of the C/L films increased with the addition of MOEO nanoemulsions (Table 2), which might be due to the light scattering at the interface of MOEO droplets embedded in the film matrix [47]. Roy and Rhim [48] reported that the addition of clove essential oil reduced the light transmittance of gelatin/agar-based functional films. In addition, the color of MOEO itself might be responsible for the decreased transparency. The low transparency of the active films improved the barrier properties of the food damaged layer (light, oxygen, heat), benefiting the long-term storage of the food [49]. Active films with lower opacity were conducive to use in light-sensitive products, and the addition of essential oils to active films could better protect food against light [50]. These active films blocked the ultraviolet light and prevented the oxidation of food, which might benefit the packaging of light-sensitive foods.

### 3.4. FTIR Results

**Figure 3:**
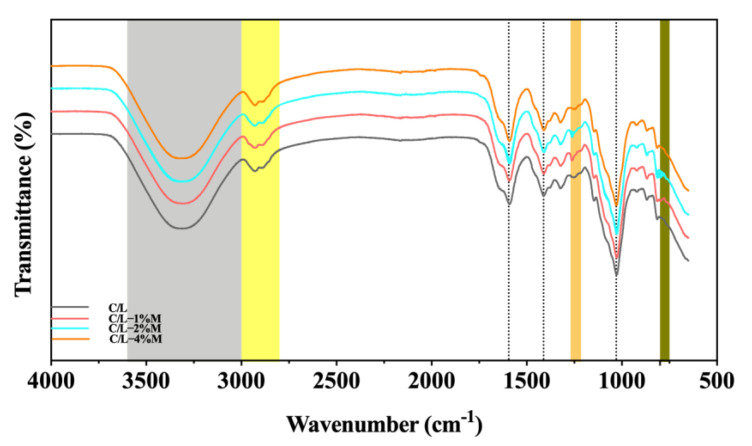
Fourier transform infrared patterns of carboxymethyl chitosan/locust bean gum and carboxymethyl chitosan/locust bean gum/*Melissa officinalis* L. essential oil films. (C/L: coated with C/L active coating solution without MOEO nano-emulsions; C/L-1%M: coated with C/L active coating solution containing 1% MOEO nano-emulsions; C/L-2%M: coated with C/L active coating solution containing 2% MOEO nano-emulsions; and C/L-4%M: coated with C/L active coating solution containing 4% MOEO nano-emulsions).

FTIR spectroscopy is an effective method to reveal the structural changes that occur through the interaction of C/L and MOEO. The band peaks in the 3600–3000 cm^−1^ range represented free OH stretching bonds and OH bonds in carboxylic acid. The stretching vibration of OH was expressed by inter-molecular or intra-molecular hydrogen bonds. The 3000–2800 cm^−1^ peaks were associated with bending and stretching vibrations of NCH- and CH_2_- [51]. The peaks at 2898 and 2851 cm^−1^ were related to the methylene and methylene C-H stretching vibrations of the biopolymer chains [48]. The active films containing MOEO nanoemulsions showed looser bands in these regions, indicating that MOEO was bound to the hydrocolloids of the film matrix (Figure 3) [30]. The band at 1600–1597 cm^−1^ was associated with COOH carboxyl groups and that at 1399–1394 cm^−1^ was associated with the CH_3_ stretching vibrations. The band at 1023–1018 cm^−1^ was caused by glycosidic bond stretching vibrations. In the second region, the bands at 1020 cm^−1^ and 921 cm^−1^ changed significantly with the increased addition of MOEO nanoemulsions because of the different types of vibration of C-O-C groups [52].

### 3.5. SEM

**Figure 4:**
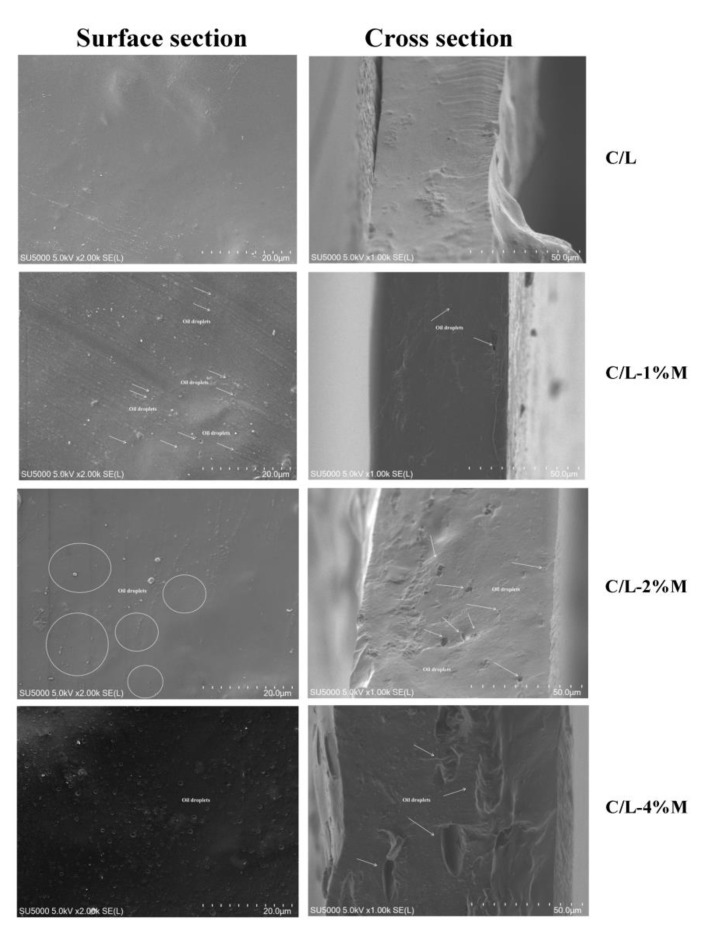
Scanning electron microscope patterns of carboxymethyl chitosan/locust bean gum and carboxymethyl chitosan/locust bean gum/*Melissa officinalis* L. essential oil films. (C/L: coated with C/L active coating solution without MOEO nano-emulsions; C/L-1%M: coated with C/L active coating solution containing 1% MOEO nano-emulsions; C/L-2%M: coated with C/L active coating solution containing 2% MOEO nano-emulsions; and C/L-4%M: coated with C/L active coating solution containing 4% MOEO nano-emulsions).

The SEM images showed the surface and cross-section of C/L film, and no holes or cracks were observed (Figure 4). The cross-section of the observed C/L-M active films had a few small oil droplets and pores in the films. The MOEO emulsions were uniformly dispersed in the active films as round oil droplets, and the absence of layering or pore structure in the films indicated that MOEO had been successfully loaded onto the films. The number of pores in the cross-section of the films gradually increased, indicating that the films were loose. In addition, the MOEO droplets were observed to increase on the cross-section and surface, and tended to be of uniform distribution. Some studies also showed similar results with the addition of cinnamaldehyde (CIN) into starch/polyvinyl alcohol (ST/PVA) films [22]. The uniform distribution of MOEO nanoemulsions indicated that the films were stable, and no separation of oil droplets occurred during the film forming process [23]. MOEO evaporated during the film formation and produced pores. Nevertheless, the films with the MOEO nanoemulsions displayed rougher surfaces with wrinkles, small cracks and some small holes. The cracks and holes on the film surface might be caused by the volatilization of essential oil on the film surface, and the coalescence, flocculation and emulsification of MOEO while drying the film, leading to the formation of some oil droplets on the film surface [53]. Xu et al. [54] and Cazón et al. [55] both revealed similar cracks and holes after incorporation of cinnamon essential oil emulsion and melaleuca alternifolia essential oil into chitosan films, respectively. The results showed that the composition of the original film-forming solutions affected the changes in film structure, namely the existence of MOEO emulsions. It was speculated that the incorporation of MOEO emulsions changed the microstructure of the active films, which could be associated with the reduction in TS and higher WVP.

### 3.6. Antioxidant and Antimicrobial Activity

**Figure 5:**
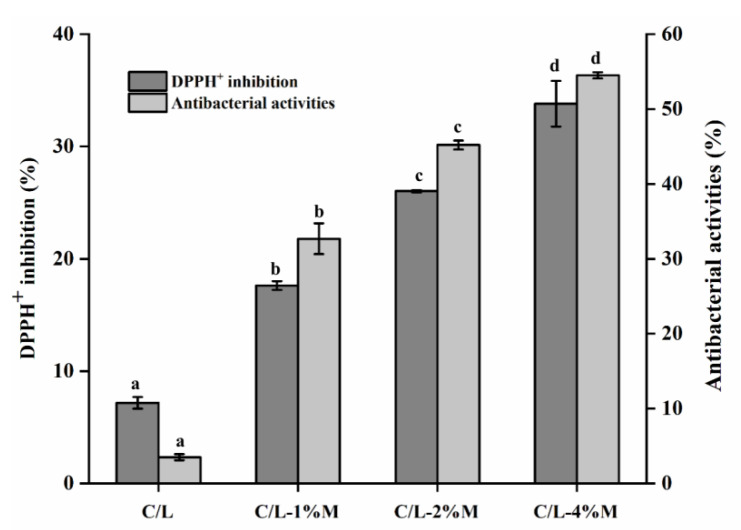
Antioxidant and antimicrobial activity of carboxymethyl chitosan/locust bean gum and carboxymethyl chitosan/locust bean gum/*Melissa officinalis L*. essential oil films. (C/L: coated with C/L active coating solution without MOEO nano-emulsions; C/L-1%M: coated with C/L active coating solution containing 1% MOEO nano-emulsions; C/L-2%M: coated with C/L active coating solution containing 2% MOEO nano-emulsions; and C/L-4%M: coated with C/L active coating solution containing 4% MOEO nano-emulsions). Different letters indicate significant differences (*p* < 0.05).

The DPPH radical scavenging activity of the films was significantly enhanced with the addition of MOEO nanoemulsion (Figure 5). The DPPH radical scavenging activity of C/L-4%M was 34.81%. C/L-4%M had the highest scavenging activity, which was nearly 5 times more than that of C/L. This phenomenon was attributed mainly to the higher antioxidant of MOEO. MOEO had numerous phenolic components and terpenes [56]. The antioxidant properties of MOEO probably were due to the presence of these substances because of their superior antioxidant activity [57].

C/L-M films also displayed excellent antibacterial activities against *S. putrefaciens*, and the antibacterial activities gradually increased with the increasing addition of MOEO nanoemulsions. The presence of numerous phenolic components such as tannins, flavonoids, and phenolic acids was responsible for the good antioxidant, antibacterial and free radical scavenging abilities of MOEO [57]. C/L films had certain antibacterial effects as CMCS had antimicrobial properties [8]. In general, it could be concluded that MOEO nanoemulsion imparted antibacterial and antioxidant activities to the C/L films, showing the potential of the films for applications in the food preservation field.

## 4. Conclusions

This research aimed to prepare C/L based active films incorporated with MOEO nanoemulsions. The results showed that the C/L–MOEO active films resulted in an increase in elongation at break, water resistance and oxygen barrier properties and improved film hydrophilicity. The ultrasonic homogenization decreased the droplet sizes of MOEO and enhanced the stability of MOEO nanoemulsions. However, the water vapor barrier properties and tensile strength of the active films decreased due to the hydrophobicity of MOEO. The SEM images and FTIR results showed that the MOEO was highly compatible with the film materials and dispersed evenly in films. At the same time, the addition of MOEO nanoemulsions significantly enhanced the antioxidant and antibacterial activities of C/L films. In general, this research provided a theoretical basis for the preparation of MOEO nanoemulsions and their application to active packaging films.

## Figures and Tables

**Table 1:** The physical and mechanical properties of carboxymethyl chitosan/locust bean gum films incorporated with *Melissa officinalis* L. essential oil nanoemulsion.

Film Samples	Thickness (cm)	Tensile Strength (MPa)	Elongation at Break (%)	Moisture Content (%)	Water Solubility (%)	Water Vapor Permeability (×10^−12^ g * cm/(cm^2^ ⋅ s ⋅ Pa))	Oxygen Transmission Rates (cm^3^/(m^2^ ⋅ 24 h ⋅ 0.1 MPa))	Water Contact Angle (θ)
C/L	0.0895 ± 0.0057 ^a^	13.44 ± 0.76 ^a^	18.49 ± 0.79 ^a^	19.59 ± 0.93 ^a^	56.32 ± 1.03 ^a^	7.47 ± 0.15 ^a^	12.43 ± 0.33 ^a^	75.13 ± 0.84 ^a^
C/L-1%M	0.0933 ± 0.0043 ^a^	5.68 ± 0.43 ^b^	25.30 ± 0.52 ^b^	19.17 ± 0.42 ^a^	34.26 ± 0.82 ^b^	8.14 ± 0.08 ^b^	8.47 ± 0.15 ^b^	78.25 ± 0.24 ^a^
C/L-2%M	0.0995 ± 0.0090 ^ab^	4.59 ± 0.53 ^b^	25.91 ± 0.58 ^b^	18.79 ± 0.42 ^a^	26.67 ± 0.43 ^c^	8.31 ± 0.20 ^c^	9.34 ± 0.31 ^c^	81.41 ± 0.44 ^a^
C/L-4%M	0.1059 ± 0.0128 ^b^	7.32 ± 0.33 ^c^	27.97 ± 0.23 ^c^	18.34 ± 0.09 ^a^	25.43 ± 0.31 ^c^	8.67 ± 0.16 ^c^	9.42 ± 0.12 ^c^	83.86 ± 0.10 ^a^

C/L (coated with C/L active coating solution without MOEO nano-emulsions); C/L-1%M (coated with C/L active coating solution containing 1% MOEO nano-emulsions); C/L-2%M (coated with C/L active coating solution containing 2% MOEO nano-emulsions); and C/L-4%M (coated with C/L active coating solution containing 4% MOEO nano-emulsions). Different letters in the same column indicate significant differences (P < 0.05).

**Table 2:** The optical properties of carboxymethyl chitosan/locust bean gum films incorporated with *Melissa officinalis* L. essential oil nanoemulsion.

Film Samples	L*	a*	b*	ΔE	Chroma	Opacity
C/L	90.06 ± 0.81 ^a^	−3.81 ± 0.11 ^a^	13.44 ± 0.35 ^a^	9.14 ± 0.89 ^a^	13.97 ± 0.34 ^a^	0.34 ± 0.02 ^a^
C/L-1%M	87.81 ± 0.70 ^b^	−4.02 ± 0.08 ^a^	16.26 ± 0.55 ^b^	12.74 ± 0.89 ^b^	16.75 ± 0.52 ^b^	0.48 ± 0.01 ^b^
C/L-2%M	88.42 ± 0.15 ^b^	−3.87 ± 0.06 ^a^	16.40 ± 0.33 ^b^	12.49 ± 0.37 ^b^	16.85 ± 0.32 ^b^	0.31 ± 0.01 ^ac^
C/L-4%M	89.56 ± 0.63 ^ab^	−3.96 ± 0.14 ^a^	16.47 ± 0.98 ^b^	11.92 ± 1.17 ^b^	16.94 ± 0.94 ^b^	0.30 ± 0.01 ^c^

C/L (coated with C/L active coating solution without MOEO nano-emulsions); C/L-1%M (coated with C/L active coating solution containing 1% MOEO nano-emulsions); C/L-2%M (coated with C/L active coating solution containing 2% MOEO nano-emulsions); and C/L-4%M (coated with C/L active coating solution containing 4% MOEO nano-emulsions). Different letters in the same column indicate significant differences (P < 0.05).

## Data Availability

Not applicable.

## References

[1] Song J., Yang W., Zhao X., Chen S., Qian G., Jiang G., Ren S., Li S. (2021). Composite films with excellent mechanical, antioxidant and UV-shielding properties prepared from oligomeric proanthocyanidin nanospheres and poly(vinyl alcohol). Ind. Crops Prod..

[2] Subramanian K., Vadivu K.S., Subramaniyam L., Kumar M.D. (2022). Synthesis, characterization, and analysis of bioplasticizer derived from Hibiscus rosa-sinensis leaves and cinnamon bark for poly (vinyl chloride) films. Ind. Crops Prod..

[3] Zou Y., Yuan C., Cui B., Wang J., Yu B., Guo L., Dong D. (2021). Mechanical and antimicrobial properties of high amylose corn starch/konjac glucomannan composite film enhanced by cinnamaldehyde/β-cyclodextrin complex. Ind. Crops Prod..

[4] Akkaya N.E., Ergun C., Saygun A., Yesilcubuk N., Akel-Sadoglu N., Kavakli I.H., Turkmen H.S., Catalgil-Giz H. (2020). New biocompatible antibacterial wound dressing candidates; agar-locust bean gum and agar-salep films. Int. J. Biol. Macromol..

[5] Singh R.S., Kaur N., Rana V., Singla R.K., Kang N., Kaur G., Kaur H., Kennedy J.F. (2020). Carbamoylethyl locust bean gum: Synthesis, characterization and evaluation of its film forming potential. Int. J. Biol. Macromol..

[6] Grala D., Biernacki K., Freire C., Kuźniarska-Biernacka I., Souza H.K.S., Gonçalves M.P. (2022). Effect of natural deep eutectic solvent and chitosan nanoparticles on physicochemical properties of locust bean gum films. Food Hydrocoll..

[7] Zhang X., Ismail B.B., Cheng H., Jin T.Z., Qian M., Arabi S.A., Liu D., Guo M. (2021). Emerging chitosan-essential oil films and coatings for food preservation—A review of advances and applications. Carbohydr. Polym..

[8] Oladzadabbasabadi N., Mohammadi Nafchi A., Ariffin F., Wijekoon M.M.J.O., Al-Hassan A.A., Dheyab M.A., Ghasemlou M. (2022). Recent advances in extraction, modification, and application of chitosan in packaging industry. Carbohydr. Polym..

[9] Jiang Y., Li D., Tu J., Zhong Y., Zhang D., Wang Z., Tao X. (2020). Mechanisms of change in gel water-holding capacity of myofibrillar proteins affected by lipid oxidation: The role of protein unfolding and cross-linking. Food Chem..

[10] de Assis R.M.A., Carneiro J.J., Medeiros A.P.R., de Carvalho A.A., da Cunha Honorato A., Carneiro M.A.C., Bertolucci S.K.V., Pinto J.E.B.P. (2020). Arbuscular mycorrhizal fungi and organic manure enhance growth and accumulation of citral, total phenols, and flavonoids in *Melissa officinalis* L. Ind. Crops Prod..

[11] Son Y.-J., Park J.-E., Kim J., Yoo G., Nho C.W. (2021). The changes in growth parameters, qualities, and chemical constituents of lemon balm (*Melissa officinalis* L.) cultivated in three different hydroponic systems. Ind. Crops Prod..

[12] Zhang W., Jiang H., Rhim J.-W., Cao J., Jiang W. (2022). Effective strategies of sustained release and retention enhancement of essential oils in active food packaging films/coatings. Food Chem..

[13] Ghosh V., Mukherjee A., Chandrasekaran N. (2014). Eugenol-loaded antimicrobial nanoemulsion preserves fruit juice against, microbial spoilage. Colloids Surf B Biointerfaces.

[14] Lomate G.B., Dandi B., Mishra S. (2018). Development of antimicrobial LDPE/Cu nanocomposite food packaging film for extended shelf life of peda. Food Packag. Shelf Life.

[15] Keawpeng I., Paulraj B., Venkatachalam K. (2022). Antioxidant and antimicrobial properties of mung bean phyto-film combined with longkong pericarp extract and sonication. Membranes.

[16] Tan M., Ding Z., Mei J., Xie J. (2022). Effect of cellobiose on the myofibrillar protein denaturation induced by pH changes during freeze-thaw cycles. Food Chem..

[17] Mei J., Yuan Y., Guo Q., Wu Y., Li Y., Yu H. (2013). Characterization and antimicrobial properties of water chestnut starch-chitosan edible films. Int. J. Biol. Macromol..

[18] Mei J., Yuan Y., Wu Y., Li Y. (2013). Characterization of edible starch–chitosan film and its application in the storage of Mongolian cheese. Int. J. Biol. Macromol..

[19] Wang Y., Nie Y., Chen C., Zhao H., Zhao Y., Jia Y., Li J., Li Z. (2022). Preparation and characterization of a thin-film composite membrane modified by mXene nano-sheets. Membranes.

[20] Agarwal C., Kóczán Z., Börcsök Z., Halász K., Pásztory Z. (2021). Valorization of Larix decidua Mill. bark by functionalizing bioextract onto chitosan films for sustainable active food packaging. Carbohydr. Polym..

[21] Rong L., Shen M., Wen H., Ren Y., Xiao W., Xie J. (2021). Preparation and characterization of hyacinth bean starch film incorporated with TiO2 nanoparticles and Mesona chinensis Benth polysaccharide. Int. J. Biol. Macromol..

[22] Chen C., Zong L., Wang J., Xie J. (2021). Microfibrillated cellulose reinforced starch/polyvinyl alcohol antimicrobial active films with controlled release behavior of cinnamaldehyde. Carbohydr. Polym..

[23] Sun H., Li S., Chen S., Wang C., Liu D., Li X. (2020). Antibacterial and antioxidant activities of sodium starch octenylsuccinate-based Pickering emulsion films incorporated with cinnamon essential oil. Int. J. Biol. Macromol..

[24] Bian C., Cheng H., Yu H., Mei J., Xie J. (2022). Effect of multi-frequency ultrasound assisted thawing on the quality of large yellow croaker *(Larimichthys crocea)*. Ultrason. Sonochem..

[25] Zhang R., Belwal T., Li L., Lin X., Xu Y., Luo Z. (2020). Recent advances in polysaccharides stabilized emulsions for encapsulation and delivery of bioactive food ingredients: A review. Carbohydr. Polym..

[26] Razavi S.M.A., Amini A.M., Zahedi Y. (2015). Characterisation of a new biodegradable edible film based on sage seed gum: Influence of plasticiser type and concentration. Food Hydrocoll..

[27] Valizadeh S., Naseri M., Babaei S., Hosseini S.M.H., Imani A. (2019). Development of bioactive composite films from chitosan and carboxymethyl cellulose using glutaraldehyde, cinnamon essential oil and oleic acid. Int. J. Biol. Macromol..

[28] Zhang Y., Zhou L., Zhang C., Show P.L., Du A., Fu J., Ashokkumar V. (2020). Preparation and characterization of curdlan/polyvinyl alcohol/ thyme essential oil blending film and its application to chilled meat preservation. Carbohydr. Polym..

[29] Otoni C.G., de Moura M.R., Aouada F.A., Camilloto G.P., Cruz R.S., Lorevice M.V., de FF Soares N., Mattoso L.H.C. (2014). Antimicrobial and physical-mechanical properties of pectin/papaya puree/cinnamaldehyde nanoemulsion edible composite films. Food Hydrocoll..

[30] Behjati J., Yazdanpanah S. (2021). Nanoemulsion and emulsion vitamin D3 fortified edible film based on quince seed gum. Carbohydr. Polym..

[31] Shen Y., Ni Z.-J., Thakur K., Zhang J.-G., Hu F., Wei Z.-J. (2021). Preparation and characterization of clove essential oil loaded nanoemulsion and pickering emulsion activated pullulan-gelatin based edible film. Int. J. Biol. Macromol..

[32] Bonilla J., Atarés L., Vargas M., Chiralt A. (2012). Effect of essential oils and homogenization conditions on properties of chitosan-based films. Food Hydrocoll..

[33] Zhou C., Li C., Siva S., Cui H., Lin L. (2021). Chemical composition, antibacterial activity and study of the interaction mechanisms of the main compounds present in the Alpinia galanga rhizomes essential oil. Ind. Crops Prod..

[34] Wu M., Zhou Z., Yang J., Zhang M., Cai F., Lu P. (2021). ZnO nanoparticles stabilized oregano essential oil Pickering emulsion for functional cellulose nanofibrils packaging films with antimicrobial and antioxidant activity. Int. J. Biol. Macromol..

[35] Chen F., Miao X., Lin Z., Xiu Y., Shi L., Zhang Q., Liang D., Lin S., He B. (2021). Disruption of metabolic function and redox homeostasis as antibacterial mechanism of Lindera glauca fruit essential oil against Shigella flexneri. Food Contr..

[36] Peng Y., Wu Y., Li Y. (2013). Development of tea extracts and chitosan composite films for active packaging materials. Int. J. Biol. Macromol..

[37] Ojagh S.M., Rezaei M., Razavi S.H., Hosseini S.M.H. (2010). Development and evaluation of a novel biodegradable film made from chitosan and cinnamon essential oil with low affinity toward water. Food Chem..

[38] Zahedi Y., Ghanbarzadeh B., Sedaghat N. (2010). Physical properties of edible emulsified films based on pistachio globulin protein and fatty acids. J. Food Eng..

[39] Ghiasi F., Golmakani M.-T., Eskandari M.H., Hosseini S.M.H. (2020). A new approach in the hydrophobic modification of polysaccharide-based edible films using structured oil nanoparticles. Ind. Crops Prod..

[40] Song X., Zuo G., Chen F. (2018). Effect of essential oil and surfactant on the physical and antimicrobial properties of corn and wheat starch films. Int. J. Biol. Macromol..

[41] Al-Harrasi A., Bhtaia S., Al-Azri M.S., Makeen H.A., Albratty M., Alhazmi H.A., Mohan S., Sharma A., Behl T. (2022). Development and characterization of chitosan and porphyran based composite edible films containing ginger essential oil. Polymers.

[42] Nogueira M.S., Scolaro B., Milne G.L., Castro I.A. (2019). Oxidation products from omega-3 and omega-6 fatty acids during a simulated shelf life of edible oils. LWT.

[43] Dong Y., Wei Z., Wang Y., Tang Q., Xue C., Huang Q. (2022). Oleogel-based Pickering emulsions stabilized by ovotransferrin–carboxymethyl chitosan nanoparticles for delivery of curcumin. LWT.

[44] Hopkins E.J., Chang C., Lam R.S.H., Nickerson M.T. (2015). Effects of flaxseed oil concentration on the performance of a soy protein isolate-based emulsion-type film. Food Res. Int..

[45] Kadam D., Shah N., Palamthodi S., Lele S.S. (2018). An investigation on the effect of polyphenolic extracts of Nigella sativa seedcake on physicochemical properties of chitosan-based films. Carbohydr. Polym..

[46] Souza V.G.L., Fernando A.L., Pires J.R.A., Rodrigues P.F., Lopes A.A.S., Fernandes F.M.B. (2017). Physical properties of chitosan films incorporated with natural antioxidants. Ind. Crops Prod..

[47] Tongnuanchan P., Benjakul S., Prodpran T. (2012). Properties and antioxidant activity of fish skin gelatin film incorporated with citrus essential oils. Food Chem..

[48] Roy S., Rhim J.-W. (2020). Preparation of carbohydrate-based functional composite films incorporated with curcumin. Food Hydrocoll..

[49] Mohammadi M., Mirabzadeh S., Shahvalizadeh R., Hamishehkar H. (2020). Development of novel active packaging films based on whey protein isolate incorporated with chitosan nanofiber and nano-formulated cinnamon oil. Int. J. Biol. Macromol..

[50] Cheng J., Wang H., Xiao F., Xia L., Li L., Jiang S. (2021). Functional effectiveness of double essential oils@yam starch/microcrystalline cellulose as active antibacterial packaging. Int. J. Biol. Macromol..

[51] Kang J., Cui S.W., Chen J., Phillips G.O., Wu Y., Wang Q. (2011). New studies on gum ghatti (*Anogeissus latifolia*) part I. Fractionation, chemical and physical characterization of the gum. Food Hydrocoll..

[52] Popescu M.-C., Dogaru B.-I., Goanta M., Timpu D. (2018). Structural and morphological evaluation of CNC reinforced PVA/Starch biodegradable films. Int. J. Biol. Macromol..

[53] Chu Y., Cheng W., Feng X., Gao C., Wu D., Meng L., Zhang Y., Tang X. (2020). Fabrication, structure and properties of pullulan-based active films incorporated with ultrasound-assisted cinnamon essential oil nanoemulsions. Food Packag. Shelf Life..

[54] Xu Y., Hou K., Gao C., Feng X., Cheng W., Wu D., Meng L., Yang Y., Shen X., Zhang Y. (2021). Characterization of chitosan film with cinnamon essential oil emulsion co-stabilized by ethyl-Nα-lauroyl-l-arginate hydrochloride and hydroxypropyl-β-cyclodextrin. Int. J. Biol. Macromol..

[55] Cazón P., Antoniewska A., Rutkowska J., Vázquez M. (2021). Evaluation of easy-removing antioxidant films of chitosan with *Melaleuca alternifolia* essential oil. Int. J. Biol. Macromol..

[56] Ilić Z.S., Milenković L., Tmušić N., Stanojević L., Stanojević J., Cvetković D. (2022). Essential oils content, composition and antioxidant activity of lemon balm, mint and sweet basil from Serbia. LWT.

[57] Adouni K., Bendif H., Zouaoui O., Kraujalis P., Flamini G., Venskutonis P.R., Achour L. (2022). Antioxidant activity of extracts obtained by high-pressure extraction procedures from Asparagus stipularis Forssk. South Afr. J. Bot..

